# Supplementary material for: Human Immunodeficiency Virus Infected Patients are Not at Higher Risk for Hepatitis E Virus Infection: A Systematic Review and Meta-Analysis
Source: Microorganisms. 2019 Nov 26;7(12):618. doi: 10.3390/microorganisms7120618 (PMC6955890; doi:10.3390/microorganisms7120618)
Supplement: Supplementary file 1 [file microorganisms-07-00618-s001.pdf]

Supplementary material

Table S1. List of excluded studies in which all the text has been evaluated.

Table S2. PRISMA 2009 Checklist.

Table 1. List of excluded studies in which all the text has been evaluated.

References	Criteria for exclusion							
	First Review				Second Review			
	i	ii	iii	iv	i	ii	iii	iv*
Abebe et al., 2017				X				X
Bivigou-Mboumba et al., 2017	X		X	X	X		X	X
Bradley-Stewart et al., 2015			X	X			X	X
Bura et al., 2017				X				
Caron et al., 2012			X	X			X	X
Crum-Cianflone et al., 2012				X				X
Đaković Rode et al., 2014			X	X			X	X
Debes et al., 2016				X				
Fainboim et al., 1999				X				
Feldt et al., 2013			X	X			X	X
Ferreira et al., 2018			X	X			X	X
Furukawa et al., 2016			X				X	
Harritshøj et al., 2018				X				X
Hassing et al., 2014			X	X			X	X
Jacobs et al., 2014				X				X
Jardi et al., 2012			X	X			X	X
Joulaei et al., 2015			X				X	
Junaid et al., 2014				X				
Kaba et al., 2011			X	X			X	X
Keane et al., 2012				X				
Kenfak-Foguena et al., 2011				X				X
Lanini et al., 2015			X	X			X	X
López-Fabal et al., 2015				X				
Madden et al., 2016				X				
Mancinelli et al., 2017			X	X			X	X
Mateos-Lindemann et al., 2012				X				X
Mateos-Lindemann et al., 2014				X				X
Maylin et al., 2012				X				
Munnéet al., 2014				X				X
Ng et al., 2000			X	X			X	X
Nouhin et al., 2015			X	X			X	X
Payne et al., 2013				X				
Pineda et al., 2014			X	X			X	X
Pischke et al., 2015			X	X			X	X
Pischke et al., 2010			X	X			X	X
Politou et al., 2015			X	X			X	X
Ramezani et al., 2013				X				
Rapicetta et al., 2013				X				
Renou et al., 2010			X	X			X	X
Riveiro-Barciela et al., 2014				X				
Rivero-Juarez et al., 2015			X	X			X	X
Scotto et al., 2014				X				X
Scotto et al., 2015				X				X
Sellier et al., 2011			X	X			X	X
Sherman et al., 2014				X				X
Shrestha et al., 2017				X				
Taha et al., 2015				X				

Yong et al., 2014			X	X			X	X
Zeng et al., 2017			X	X			X	X
Zhou et al., 2018			X	X			X	X

Legend: i) Articles written in English; ii) Studies evaluating anti-HEV IgG by ELISA in HIV- infected patients; iii) Studies comparing HEV seroprevalence between HIV patients and a control group; iv) Studies with a control group matched by sex, age and area.

*Studies with control group matched only by geographical area.

Table references

Abebe M, Ali I, Ayele S, Overbo J, Aseffa A, Mihret A. Seroprevalence and risk factors of Hepatitis E Virus infection among pregnant women in Addis Ababa, Ethiopia. PLoS One (2017) 9; 12(6):e0180078

Bivigou-Mboumba B, Rouet F, Mouinga-Ondeme A, Deleplancque L, Sica J, Ndjoyi-Mbiguino A, et al. Hepatitis B, C, and E infection among HIV-infected patients in Franceville, Gabon: retrospective cross-sectional study. Medecine et santetropicales (2017) 27(3):274-280

Bradley-Stewart AJ, Jesudason N, Michie K, Winter AJ, Gunson RN. Hepatitis E in Scotland: assessment of HEV infection in two high-risk patient groups with elevated liver enzymes. Journal of Clinical Virology (2015) 63:36-7

Bura M, Bukowska A, Bura A, Michalak M, Mozer-Lisewska I. Hepatitis E virus antibodies in HIV-infected patients and blood donors from western Poland: A preliminary report. Advances in Clinical and Experimental Medicine (2017) 26(4):577-579

Caron M, Bouscaillou J, Kazanji M. Acute risk for hepatitis E virus infection among HIV-1-positive pregnant women in central Africa. Journal of Virology (2012) 31;9:254

Crum-Cianflone NF, Curry J, Drobeniuc J, Weintrob A, Landrum M, Ganesan A, et al. Hepatitis E virus infection in HIV-infected persons. Emerging Infectous Diseases (2012) 18(3):502-6

Đaković Rode O, Jemeršić L, Brnić D, Pandak N, Mikulić R, Begovac J, et al. Hepatitis E in patients with hepatic disorders and HIV-infected patients in Croatia: is one diagnostic method enough for hepatitis E diagnosis? European Journal of Clinical Microbiology & Infectious Diseases (2014) 33(12):2231-6

Debes JD, Martínez Wassaf M, Pisano MB, Isa MB, Lotto M, Marianelli LG, et al. Increased Hepatitis E Virus Seroprevalence Correlates with Lower CD4+ Cell Counts in HIV-Infected Persons in Argentina. PLoSOne (2016) 11(7):e0160082

Fainboim H, González J, Fassio E, Martínez A, Otegui L, Eposto M, et al. Prevalence of hepatitis viruses in an anti-human immunodeficiency virus-positive population from Argentina. A multicentre study. Journal of Viral Hepatitis (1999) 6(1):53-7

Feldt T, Sarfo FS, Zoufaly A, Phillips RO, Burchard G, van Lunzen J, et al. Hepatitis E virus infections in HIV-infected patients in Ghana and Cameroon. Journal of Clinical Virology (2013) 58(1):18-23

Ferreira AC, Gomes-Gouvêa MS, Lisboa-Neto G, Mendes-Correa MCJ, Picone CM, Salles NA, et al. Serological and molecular markers of hepatitis E virus infection in HIV-infected patients in Brazil. Archives of Virology (2018) 163(1):43-49

Furukawa NW, Teshale EH, Cosmas L, Ochieng M, Gikunju S, Fields BS, et al. Serologic evidence for hepatitis E virus infection among patients with undifferentiated acute febrile illness in Kibera, Kenya. Journal of Clinical Virology (2016) 77:106-8

Hassing RJ, van der Eijk AA, Lopes VB, Snijdewind IJ, de Man RA, Pas SD, et al. Hepatitis E prevalence among HIV infected patients with elevated liver enzymes in the Netherlands. Journal of Clinical Virology (2014) 60(4):408-10

Harritshøj LH, Theilgaard ZP, Mannheimer E, Midgley SE, Chiduo M, Ullum H, et al. Hepatitis E virus epidemiology among HIV-infected women in an urban area in Tanzania. International Journal of Infectious Diseases (2018) 73:7-9.

Jacobs C, Chiluba C, Phiri C, Lisulo MM, Chomba M, Hill PC, et al. Seroepidemiology of hepatitis E virus infection in an urban population in Zambia: strong association with HIV and environmental enteropathy. Journal of InfectiousDiseases (2014) 209(5):652-7

Jardi R, Crespo M, Homs M, van den Eynde E, Girones R, Rodriguez-Manzano J, et al. HIV, HEV and cirrhosis: evidence of a possible link from eastern Spain. HIV Medicine (2012) 13(6):379-83

Joulaei H, Rudgari O, Motazedian N, Gorji-Makhsous S. Hepatitis E virus seroprevalence in HIV positive individuals in Shiraz, Southern Iran. Iranian Journal of Microbiology (2015) 7(2):103-8

Junaid SA, Agina SE, Abubakar KA. Epidemiology and associated risk factors of hepatitis e virus infection in plateau state, Nigeria. Virology (Auckland) (2014) 5:15-26

Kaba M, Richet H, Ravaux I, Moreau J, Poizot-Martin I, Motte A, et al. Hepatitis E virus infection in patients infected with the human immunodeficiency virus. Journal of Medical Virology (2011) 83(10):1704-16

Keane F, Gompels M, Bendall R, Drayton R, Jennings L, Black J, et al. Hepatitis E virus coinfection in patients with HIV infection. HIV Medicine (2012) 13(1):83-8

Kenfak-Foguena A, Schöni-Affolter F, Bürgisser P, Witteck A, Darling KE, Kovari H, et al. Hepatitis E Virus seroprevalence and chronic infections in patients with HIV, Switzerland. Emerging Infectious Diseases (2011) 17(6):1074-8

Lanini S, Garbuglia AR, Lapa D, Puro V, Navarra A, Pergola C, et al. Epidemiology of HEV in the Mediterranean basin: 10-year prevalence in Italy. BMJ Open (2015) 5(7):e007110

López-Fabal MF, &Gómez-Garcés JL. Seroprevalence of hepatitis E virus in patients with hepatitis C and / or infected with HIV. Revista Española de Quimioterapia (2015) 28(6):314-6

Madden RG, Wallace S, Sonderup M, Korsman S, Chivese T, Gavine B, et al. Hepatitis E virus: Western Cape, South Africa. World Journal of Gastroenterology (2016) 22(44):9853-9859

Mancinelli S, Pirillo MF, Liotta G, Andreotti M, Jere H, Sagno JB, et al. Hepatitis E virus infection in HIV-infected pregnant women and their children in Malawi. InfectiousDiseases (London) (2017) 49(9):708-711

Mateos-Lindemann ML, Diez-Aguilar M, Galdamez AL, Galán JC, Moreno A, Pérez-Gracia MT. Patients infected with HIV are at high-risk for hepatitis E virus infection in Spain. Journal of Medical Virology (2014) 86(1):71-4

Mateos-Lindemann ML, Gonzalez-Galdámez A, Bordallo-Cardona M, Pérez-Gracia MT. Are HIV-infected patients a high-risk population for hepatitis E virus infection in Spain? Enfermedades Infecciosas y Microbiología Clinica (2012) 30(9):582-3

Maylin S, Stephan R, Molina JM, Peraldi MN, Scieux C, Nicand E, et al. Prevalence of antibodies and RNA genome of hepatitis E virus in a cohort of French immunocompromised. Journal of Clinical Virology (2012) 53(4):346-9

Munné MS, Altabert NR, Otegui MLO, Vladimirsky SN, Moreiro R, Espul MP, et al. Updating the knowledge of hepatitis E: new variants and higher prevalence of anti-HEV in Argentina. Annals of Hepatology (2014) 13(5):496-502

Ng KP, He J, Saw TL, Lyles CM. A seroprevalence study of viral hepatitis E infection in human immunodeficiency virus type 1 infected subjects in Malaysia. Medical Journal of Malaysia (2000) 55(1):58-64

Nouhin J, Barennes H, Madec Y, Prak S, Hou SV, Kerleguer A, et al. Low frequency of acute hepatitis E virus (HEV) infections but high past HEV exposure in subjects from Cambodia with mild liver enzyme elevations, unexplained fever or immunodeficiency due to HIV-1 infection. Journal of Clinical Virology (2015) 71:22-7

Payne BA, Medhi M, Ijaz S, Valappil M, Savage EJ, Gill ON, et al. Hepatitis E virus seroprevalence among men who have sex with men, United Kingdom. Emerging Infectious Diseases (2013) 19(2):333-5

Pineda JA, Cifuentes C, Parra M, Merchante N, Pérez-Navarro E, Rivero-Juárez A, et al. Incidence and natural history of hepatitis E virus coinfection among HIV-infected patients. AIDS (2014) 28(13):1931-7

Pischke S, Ho H, Urbanek F, Meyer-Olsen D, Suneetha PV, Manns MP, et al. Hepatitis E in HIV-positive patients in a low-endemic country. Journal of Viral Hepatitis (2010) 17(8):598-9

Pischke S, Schwarze-Zander C, Bremer B, Lehmann P, Wiegand SB, Gisa A, et al. Hepatitis E Virus Seroprevalence Rate in HIV-Infected Patients in Germany: A Comparison of Two Commercial Assays. Intervirology (2015) 58(5):283-7

Politou M, Boti S, Androutsakos T, Valsami S, Pittaras T, Kapsimali V. Seroprevalence of hepatitis E in HIV infected patients in Greece. Journal of Medical Virology (2015) 87(9):1517-20

Rapicetta M, Monarca R, Kondili LA, Chionne P, Madonna E, Madeddu G, et al. Hepatitis E virus and hepatitis A virus exposure in an apparently healthy high-risk population in Italy. Infection (2013) 41(1):69-76

Ramezani A, Velayati AA, Khorami-Sarvestani S, Eslamifar A, Mohraz M, Banifazl, M, et al. Hepatitis E virus infection in patients infected with human immunodeficiency virus in an endemic area in Iran. International Journal of STD & AIDS (2013) 24(10):769-74

Renou C, Lafeuillade A, Cadranel JF, Pavio N, Pariente A, Allègre T, et al. Hepatitis E virus in HIV-infectedpatients. AIDS (2010) 24(10):1493-9

Riveiro-Barciela M, Buti M, Homs M, Campos-Varela I, Cantarell C, Crespo M, et al. Cirrhosis, liver transplantation and HIV infection are risk factors associated with hepatitis E virus infection. PLoSOne (2014) 9(7):e103028

Rivero-Juarez A, Martinez-Dueñas L, Martinez-Peinado A, Camacho A, Cifuentes C, Gordon A, et al. High hepatitis E virus seroprevalence with absence of chronic infection in HIV-infected patients. Journal of Infection (2015) 70(6):624-30

Scotto G, Grisorio B, Filippini P, Ferrara S, Massa S, Bulla F, et al. Hepatitis E virus co-infection in HIV-infected patients in Foggia and Naples in southern Italy. Infectious Diseases (London) (2015) 47(10):707-13.

Scotto G, Martinelli D, Centra M, Querques M, Vittorio F, Delli Carri P, et al. Epidemiological and clinical features of HEV infection: a survey in the district of Foggia (Apulia, Southern Italy). Epidemiology and Infection (2014) 142(2):287-94

Sellier P, Mazeron MC, Tesse S, Badsi E, Evans J, Magnier JD, et al. Hepatitis E virus infection in HIV-infected patients with elevated serum transaminases levels. Journal of Virology (2011) 8:171

Sherman KE, Terrault N, Barin B, Rouster SD, Shata MT, HIV-TR Investigators. Hepatitis E infection in HIV-infected liver and kidney transplant candidates. Journal of Viral Hepatitis (2014) 21(8):e74-7

Shrestha A, Adhikari A, Bhattarai M, Rauniyar R, Debes JD, Boonstra A, et al. Prevalence and risk of hepatitis E virus infection in the HIV population of Nepal. Journal of Virology (2017) 14(1):228

Taha TE, Rusie LK, Labrique A, Nyirenda M, Soko D, Kamanga M, et al. Seroprevalence for Hepatitis E and Other Viral Hepatitides among Diverse Populations, Malawi. Emerging Infectious Diseases (2015) 21(7):1174-82

Yong MK, Paige EK, Anderson D, Hoy JF. Hepatitis E in Australian HIV-infected patients: an under-recognised pathogen? Sex Health (2014) 11(4):375-8

Zeng H, Wang L, Liu P, Liao L, Wang L, Shao, Y. Seroprevalence of hepatitis E virus in HIV-infected patients in China. AIDS (2017) 31(14):2019-2021

Zhou S, Ren L, Xia X, Miao Z, Huang F, Li Y, et al. Hepatitis E virus infection in HIV-infected patients: A large cohort study in Yunnan province, China. Journal of Medical Virology (2018) 90(6):1121-1127

PRISMA 2009 Checklist

Section/topic	#	Checklist item	Reported on page #
**TITLE**			
Title	1	Identify the report as a systematic review, meta-analysis, or both.	1
**ABSTRACT**			
Structured summary	2	Provide a structured summary including, as applicable: background; objectives; data sources; study eligibility criteria, participants, and interventions; study appraisal and synthesis methods; results; limitations; conclusions and implications of key findings; systematic review registration number.	4
**INTRODUCTION**			
Rationale	3	Describe the rationale for the review in the context of what is already known.	5
Objectives	4	Provide an explicit statement of questions being addressed with reference to participants, interventions, comparisons, outcomes, and study design (PICOS).	5
**METHODS**			
Protocol and registration	5	Indicate if a review protocol exists, if and where it can be accessed (e.g., Web address), and, if available, provide registration information including registration number.	-
Eligibility criteria	6	Specify study characteristics (e.g., PICOS, length of follow-up) and report characteristics (e.g., years considered, language, publication status) used as criteria for eligibility, giving rationale.	6
Information sources	7	Describe all information sources (e.g., databases with dates of coverage, contact with study authors to identify additional studies) in the search and date last searched.	6
Search	8	Present full electronic search strategy for at least one database, including any limits used, such that it could be repeated.	6
Study selection	9	State the process for selecting studies (i.e., screening, eligibility, included in systematic review, and, if applicable, included in the meta-analysis).	6
Data collection process	10	Describe method of data extraction from reports (e.g., piloted forms, independently, in duplicate) and any processes for obtaining and confirming data from investigators.	6
Data items	11	List and define all variables for which data were sought (e.g., PICOS, funding sources) and any assumptions and simplifications made.	6-7
Risk of bias in individual studies	12	Describe methods used for assessing risk of bias of individual studies (including specification of whether this was done at the study or outcome level), and how this information is to be used in any data synthesis.	7
Summary measures	13	State the principal summary measures (e.g., risk ratio, difference in means).	7
Synthesis of results	14	Describe the methods of handling data and combining results of studies, if done, including measures of consistency (e.g., I^2) for each meta-analysis.	7

PRISMA 2009 Checklist

Section/topic	#	Checklist item	Reported on page #
Risk of bias across studies	15	Specify any assessment of risk of bias that may affect the cumulative evidence (e.g., publication bias, selective reporting within studies).	7-8
Additional analyses	16	Describe methods of additional analyses (e.g., sensitivity or subgroup analyses, meta-regression), if done, indicating which were pre-specified.	-
**RESULTS**			
Study selection	17	Give numbers of studies screened, assessed for eligibility, and included in the review, with reasons for exclusions at each stage, ideally with a flow diagram.	8-9
Study characteristics	18	For each study, present characteristics for which data were extracted (e.g., study size, PICOS, follow-up period) and provide the citations.	8-9
Risk of bias within studies	19	Present data on risk of bias of each study and, if available, any outcome level assessment (see item 12).	8-9
Results of individual studies	20	For all outcomes considered (benefits or harms), present, for each study: (a) simple summary data for each intervention group (b) effect estimates and confidence intervals, ideally with a forest plot.	9
Synthesis of results	21	Present results of each meta-analysis done, including confidence intervals and measures of consistency.	9
Risk of bias across studies	22	Present results of any assessment of risk of bias across studies (see Item 15).	9-10
Additional analysis	23	Give results of additional analyses, if done (e.g., sensitivity or subgroup analyses, meta-regression [see Item 16]).	-
**DISCUSSION**			
Summary of evidence	24	Summarize the main findings including the strength of evidence for each main outcome; consider their relevance to key groups (e.g., healthcare providers, users, and policy makers).	10
Limitations	25	Discuss limitations at study and outcome level (e.g., risk of bias), and at review-level (e.g., incomplete retrieval of identified research, reporting bias).	10-11
Conclusions	26	Provide a general interpretation of the results in the context of other evidence, and implications for future research.	11
**FUNDING**			
Funding	27	Describe sources of funding for the systematic review and other support (e.g., supply of data); role of funders for the systematic review.	3

From: Moher D, Liberati A, Tetzlaff J, Altman DG, The PRISMA Group (2009). Preferred Reporting Items for Systematic Reviews and Meta-Analyses: The PRISMA Statement. PLoS Med 6(7): e1000097. doi:10.1371/journal.pmed1000097

For more information, visit: **www.prisma-statement.org**.
